# Single-Cell Sequencing Reveals Differential Cell Types in Skin Tissues of Liaoning Cashmere Goats and Key Genes Related Potentially to the Fineness of Cashmere Fiber

**DOI:** 10.3389/fgene.2021.726670

**Published:** 2021-11-10

**Authors:** Zeying Wang, Yanru Wang, Taiyu Hui, Rui Chen, Yanan Xu, Yu Zhang, He Tian, Wei Wang, Yuyan Cong, Suping Guo, Yanxu Zhu, Xinghui Zhang, Dan Guo, Man Bai, Yixing Fan, Chang Yue, Zhixian Bai, Jiaming Sun, Weidong Cai, Xinjiang Zhang, Ming Gu, Yuting Qin, Yinggang Sun, Yanzhi Wu, Rina Wu, Xingtang Dou, Wenlin Bai, Yuanyuan Zheng

**Affiliations:** ^1^College of Animal Science and Veterinary Medicine, Shenyang Agricultural University, Shenyang, China; ^2^College of Food Science, Shenyang Agricultural University, Shenyang, China; ^3^Liaoning Province Modern Agricultural Production Base Construction Engineering Center, Shenyang, China; ^4^Liaoning Provincial Department of Science and Technology, Shenyang, China

**Keywords:** 10 × Genomics single-cell sequencing, cashmere fineness, SDPCs, *COL1A1*, *ACTA2*, *CXCL8*

## Abstract

Cashmere fineness is one of the important factors determining cashmere quality; however, our understanding of the regulation of cashmere fineness at the cellular level is limited. Here, we used single-cell RNA sequencing and computational models to identify 13 skin cell types in Liaoning cashmere goats. We also analyzed the molecular changes in the development process by cell trajectory analysis and revealed the maturation process in the gene expression profile in Liaoning cashmere goats. Weighted gene co-expression network analysis explored hub genes in cell clusters related to cashmere formation. Secondary hair follicle dermal papilla cells (SDPCs) play an important role in the growth and density of cashmere. *ACTA2*, a marker gene of SDPCs, was selected for immunofluorescence (IF) and Western blot (WB) verification. Our results indicate that ACTA2 is mainly expressed in SDPCs, and WB results show different expression levels. *COL1A1* is a highly expressed gene in SDPCs, which was verified by IF and WB. We then selected *CXCL8* of SDPCs to verify and prove the differential expression in the coarse and fine types of Liaoning cashmere goats. Therefore, the *CXCL8* gene may regulate cashmere fineness. These genes may be involved in regulating the fineness of cashmere in goat SDPCs; our research provides new insights into the mechanism of cashmere growth and fineness regulation by cells.

## Introduction

Cashmere goats are a species with high economic value. Cashmere has a certain position in the international market, and their quality meets the requirements. Among them, the value of cashmere is the most important. Cashmere includes two parts, the primary and secondary hair follicles, and the growth of cashmere is regulated by secondary hair follicles and is the main source of cashmere (Bai et al., 2018). Cashmere is the unmedullated villous fiber growing from the secondary hair follicles of goat skin, which resides in the underlayer of the coat. The secondary hair follicles of cashmere goats show regular periodic growth, which generally goes through three stages: growth, regressive, and resting.

The growth cycle of secondary hair follicles of cashmere goats is mainly affected by the length of light, temperature, feeding conditions, and breed; as a result, it presents seasonal changes (Wang et al., 2017). Although the output of cashmere is high, the overall trend of cashmere is coarse, affecting the formation of textile crafts.

We detect genetics and backgrounds that affect cashmere fineness from different aspects. Liu et al. detect candidate genes that are closely related to cashmere fineness in Inner Mongolia cashmere goats by genome-wide association analysis (Liu et al., 2012). *KAP* and its family are an important element that is shown to regulate the fineness of cashmere (Jin et al., 2017). To find a more reliable internal genetic background that determines the fineness of cashmere, we use the most advanced single-cell RNA-seq, a technique for observing gene expression at the single-cell level (Gong et al., 2018). Single-cell sequencing enables more precise selection of key genes involved in cashmere fineness in secondary hair follicle dermal papilla cells (SDPCs). Through this, we can determine the true nature of certain specific genes that can be expressed in secondary hair follicle cells. The latest single-cell RNA sequencing (scRNA-seq) is able to identify a large number of cells (Buenrostro et al., 2018; Pace et al., 2018; Pandey et al., 2018; Shah et al., 2018). In mice, small intestine single-cell sequencing was used to obtain helper cell types that support intestinal stem cells to produce mature epithelial cell types (Biton et al., 2018). Similarly, single-cell transcriptomic analysis reveals heterogeneous differentiation and spatial characteristics of mouse epidermis and hair follicles (Joost et al., 2016). Furthermore, to determine the differences in oocyte maturation at different stages, transcriptome sequencing of single cells was compared with oocyte maturation gene expression in children and female goats to screen and verify genes related to oocyte maturation (Yin et al., 2017). However, research on regulating the fineness of cashmere at the cellular level is still scarce.

Herein, the purpose of this study was to investigate cell-/tissue-specific transcriptional profiles. Therefore, the cells of cashmere goat skin were sequenced using single-cell sequencing technology to systematically analyze the cell heterogeneity in tissue-stable epidermal cells. We employed cell trajectory analysis to reveal molecular changes during development, and used immunofluorescence (IF) and Western blot (WB) to verify new key genes that may be a turning point in regulating the fineness of cashmere. Through deep research and analysis of cashmere skin cells, more gene information can be obtained to screen the genes related to cashmere fiber fineness more clearly and accurately so as to improve the quality of cashmere, increase the economic benefit, and lay the foundation for the future research on cashmere fiber fineness correlation. Our original data is in the GEO database, and the GSE accession ID is GSE182474.

## Results and Analyses

### Single-Cell Sequencing Identifies 13 Distinct Cell Populations in Goat Skin

**FIGURE 1:**
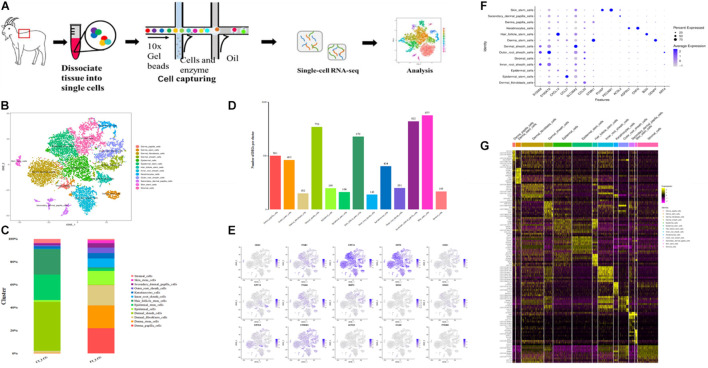
Identification of cashmere goat skin cell types. **(A)** Schematic representation of cashmere goat skin tissue preparation for single-cell transcriptome analysis. **(B)** t-SNE clustering of cells based on expression levels of genes. Each color represents a class of cells. **(C)** Graph showing percentage of each cluster in two samples. **(D)** Number of DEGs in each cluster. **(E)** t-SNE plots of marker genes expression value in per cluster. **(F)** Highly expressed genes in every cell type. **(G)** Heat map of the top 10 specific marker genes.

To better define the cell types in Liaoning cashmere goat skin, we performed a scRNA-seq analysis in the coarse (CT-LCG) and fine (FT-LCG) types of Liaoning cashmere goat skin in their secondary hair follicle stage of prime growth. For every type of goat, we obtained 551,655,085 and 556,307,136 reads. The sequencing saturation rates were 76.9 and 68.3%, respectively, (Figure 1A). We separated and sequenced 6,102 and 11,197 cells, following strict quality control; 3,443 and 9,608 cells remained for downstream analysis in CT-LCG and FT-LCG.

We first defined the different cell populations in cashmere goat skin, using the analysis of unsupervised clustering and *t-*distributed stochastic neighbor embedding dimension reduction (t-SNE). After standard quality control excluded cells including gene check <500 and mitochondrial gene coverage >10%, samples underwent detailed clustering analysis by Seurat workflow to define 13 distinct populations (Figure 1B). The average expression profile of the two samples is highly correlated, and the number of cells in each type is shown in Figure 1C. We found that dermal sheath cells, epidermal stem cells, and hair follicle stem cells make up a large proportion of CT-LCG; meanwhile, dermal papilla cells (DPCs), dermal stem cells, dermal fibroblast cells, and epidermal cells are the largest component of FT-LCG. We identified the gene expression profile per cluster; differential expression genes (DEGs) refer to the difference between the average expression of cells in clusters (Figure 1D). The top 10 up-expression genes of each cluster are shown in Table 1. In total, two kinds of DPCs, four stem cells, three sheath cells, one keratinocyte, one fibroblast, one epidermal cell, and one stromal cell were identified as assigned in goat skin. We detected that genes enriched in distinct clusters differently marked various cell types. For instance, *KRT14* and *KRT5* are known expression markers in epidermis stem cells (Cluster 1; Figure 1E). *DKK1* are highly expressed in epidermis cells (Cluster 2). Dermal sheath cells (Cluster 6) were identified with a specific expression of known marker gene *BMP2.* Genes enriched in Cluster 7 were strongly labeled in dermal stem cells and expressed cell markers, such as *SOX2* and *FOXM1.* Hair follicle stem cells (Cluster 8, HFSCs) promote hair development and were identified by multiple specific markers (e.g., *KRT19; DSG3;* and *ITGA6*). Skin stem cells (Cluster 12) also expressed selective genes, including *CD34* and *ITGB1*. Finally, we found that cells from C10 showed a characteristic of DPCs, which is a high expression level of *CTNNB1*, and C11 showed characteristics of SDPCs, such as high expression levels of *VCAN* and *ACTA2.* Moreover, dermal fibroblast cells markers (e.g., *VUM* and *COL1A1*), inner root sheath cell markers (e.g., *CDIP1; CDS1;* and *FOXN1*), outer root sheath cell markers (e.g., *DOCK6* and *KRT19*), a stromal cell marker (e.g., *ITGA6*), and a keratinocyte cell marker (e.g., *KRT6A*) showed specificity expression in Clusters 0, 3, 5, 4, and 9, respectively.

**TABLE 1:** The top 10 genes per clusters.

**Expression**	Cluster 0	Cluster 1	Cluster 2	Cluster 3	Cluster 4	Cluster 5	Cluster 6	Cluster 7	Cluster 8	Cluster 9	Cluster 10	Cluster 11	Cluster 12
Top1	KRT10	KRT14	KRT14	LOC108634769	KRT10	KRT10	KRT1	KRT14	KRT5	CST6	RPS12	LOC108634769	TMSB4X
	148.51	334.64	170.19	232.43	232.68	322.73	197.52	194.81	125.12	792.65	132.77	82.62	102.97
Top2	KRT14	KRT5	KRT5	KRT10	LOC102177275	KRT1	KRT10	KRT5	KRT14	KRTDAP	LOC102168701	LOC102168701	LOC108634769
	127.88	158.27	129.94	173.85	179.90	197.82	155.68	112.26	112.85	300.61	118.94	73.91	97.50
Top3	LOC102177275	LGALS7	LOC102177275	KRT1	KRT1	S100A7A	LOC108634769	PTMA	LOC102179515	KRT10	RPS8	COL1A1	VIM
	108.49	108.19	110.33	128.36	132.21	155.10	116.21	95.76	106.09	265.48	115.23	71.50	97.21
Top4	CST6	LOC102168701	RPS8	RPLP1	LGALS7	KRTDAP	RPLP1	RPS8	LOC102168701	SBSN	RPLP1	RPS8	TPT1.1
	101.48	105.19	94.81	93.46	110.19	99.43	71.73	90.80	93.90	174.83	97.81	70.63	84.12
Top5	KRT5	TPT1.1	RPLP1	LOC102168701	ACTG1	SPINK7	LOC108635081	LOC102168701	RPS8	DMKN	RPLP0	VIM	LOC102168701
	88.78	101.40	80.37	90.56	103.55	97.56	70.77	87.07	91.65	109.14	87.65	62.30	80.81
Top6	ACTG1	RPLP1	LOC102168701	RPS8	CST6	LGALS7	LGALS7	RPLP1	RPS12	KRT1	RPS2	RPLP1	RPS8
	81.70	98.20	79.72	87.21	88.81	86.67	66.77	85.31	83.15	107.72	80.94	61.71	78.59
Top7	KRT1	RPS8	TPT1.1	RPS12	KRTDAP	RPLP1	JUN	RPS12	TPT1.1	HSPB1	RPL32	S100A4	LOC102182395
	70.62	91.93	70.67	80.54	84.74	83.42	61.55	67.79	79.14	89.67	76.40	61.11	75.92
Top8	LGALS7	S100A2	RPS12	S100A7A	RPS8	LOC102168701	KRTDAP	LGALS7	RPLP1	LGALS7	LOC102184223	RPS12	RPS12
	70.56	90.87	68.12	67.47	80.17	79.57	58.47	66.43	76.50	81.33	75.60	60.18	70.72
Top9	LOC102168701	RPS12	LGALS7	LGALS7	HSPB1	SBSN	RPS8	RPS19	LOC102177275	LOC102177275	RPS5	TPT1.1	LOC108635081
	67.65	84.82	66.98	66.95	75.44	76.47	55.76	64.08	73.90	80.80	74.67	57.61	67.08
Top10	RPLP1	RPS19	RPS19	LOC102169125	RPLP1	RPS12	UBB	RPS2	RPS19	RPLP1	RPL31	RPS24	RPLP1
	66.06	80.19	65.51	64.82	74.39	70.96	55.69	60.15	72.41	70.05	73.46	50.28	66.72

Importantly, the high-expression genes per cell group are shown in Figure 1F. *KRT5* and *KRT14* were expressed highest in epidermis stem cells and significantly enriched in Clusters 0, 2, 7, and 8. *CCL27*, *ADIRF*, and *LY6D* were obviously differentially expressed in epidermis stem cells. *KRT10* were expressed highest in Clusters 4 and 5. *LOC102179515*, *NFIB*, *BGN*, and *SFPRP1* were overexpressed on Cluster 8. Interestingly, *CXCL14* was overexpressed on Clusters 8 and 11, which may be related to hair development. Indeed, *KRTDAP* and *CST6* were expressed highest in Cluster 9. Notably, *KRT35* and *KRTAP11-1* may be related to cashmere character (Zheng et al., 2019), whereas, in goats, their expressions are confined to DPCs. In addition, *LEF1* was considered to be a hair-inducing gene of DPCs (Dong et al., 2017). The expression of the positive genes *IGFBP5* and *IGFBP6* is discovered highly expressed in SDPCs. *ALX4*, which may be related to cashmere fineness (Qiao et al., 2017), is only expressed in DPCs and SDPCs. *CCL21*, *GNG11*, and *TFPI2* are expressed highest in HFSCs, and *VIM* and *CLEC3B* were overexpressed on DPCs and HFSCs. *KRT19* and *SOX9* were proven to exist in the anagen secondary HFSCs (He et al., 2020), which are highly expressed in our results in HFSCs. Some genes are only expressed in certain cells, such as *PLVAP*, *PECAM1*, and *CENPF*. These significantly up-expressed genes, especially overexpressed in each functional cluster, and may be functional candidate genes associated with fibrogenesis. Collectively, these data establish the classification of skin cells and the selective gene expression pattern of cashmere goat. Below is an overview of DEGs. There is the heat map of the top 10 specific marker genes in Figure 1G, which showed analysis results of differentially expressed genes.

### Cell Trajectory Analysis Reveals Cell Development in Cashmere Goat Skin

To further investigate the classification of skin cell populations and their developmental relationship, we performed cell trajectory analysis using the Monocle analysis toolkit (Ming and Song, 2011). To enable a single cell to differentially express genes during development based on their trajectories, cells were calculated by Monocle 2 in an unsupervised way via maximizing transcription similarity between consecutive cell pairs. Therefore, this method can be used to identify various phases of skin development in cashmere goats. Monocyte 2 had a large overlap in each cluster, and the subpopulation of cell classification was distributed in the whole skin, indicating versatility.

**FIGURE 2:**
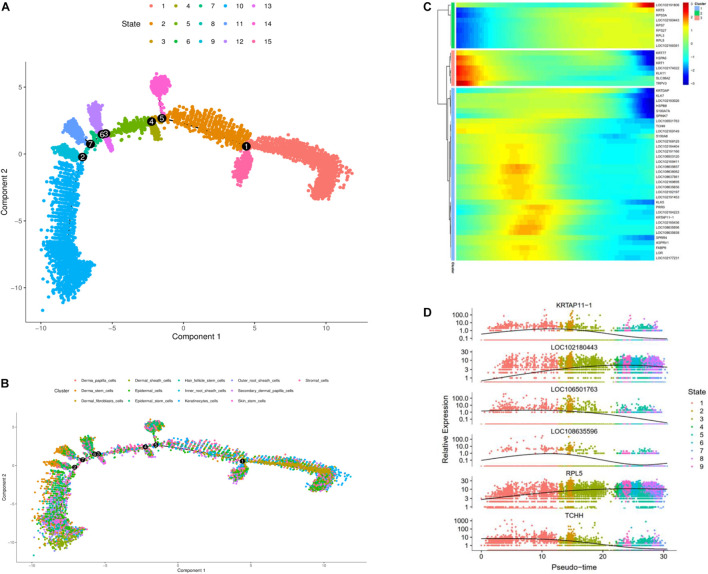
Cell trajectory analysis on goat skin cells. **(A)** Monocle 2 cell trajectory analysis of goat skin cells. **(B)** Monocle 2 cell trajectory colored according to the identity of 13 goat skin cells. **(C)** Clustering of genes represent differential expression patterns across goat skin cell populations. **(D)** Dynamic expression values of top six different genes are plotted on pseudo-time. *X*-axis represents pseudo time, and *Y*-axis represents gene expression levels.

To explore which gene regulates cashmere fiber development progression per axis, we classified and clustered the gene expression changes with cell trajectory. Cell trajectory analysis results revealed the skin development of the cashmere goat is divided into 15 states, and every kind of cell was involved in almost the whole development process (Figures 2A,B). In support of the draw order in cell trajectory, our analysis revealed the organization of genes that are differentially expressed in each branch, which is closely related to the genes that are famous for participating in cashmere growth and hair development. Interestingly, *KRTAP11-1*, *TCHH*, *KRTDAP*, and *S100A8* that were briefly up-regulated and then down-regulated were observed. However, a subset of genes, such as *KRT5*, *RPS7*, *RPL3*, and *RPL5*, were sustained as up-regulated over time (Figure 2C). Further confirming the validity of the cell trajectory analysis, the top 6 genes, such as *KRTAP11-1* and *TCHH* (Figure 2D), which GO analysis enriched with intermediate filament and keratinization, are important pathways for cashmere growth, indicating these genes may relate to cashmere formation and development. KEGG pathways of these genes mainly enriched to the WNT, TGF beta, and PI3K-Akt signaling pathways. These data and analyses reveal the clear developmental stages of goat skin; the expressions of differential genes along the timeline of bifurcations indicates that different molecular paths guide the development of different populations.

### Weighted Gene Correlation Network Analysis of Cashmere Goat Skin

**FIGURE 3:**
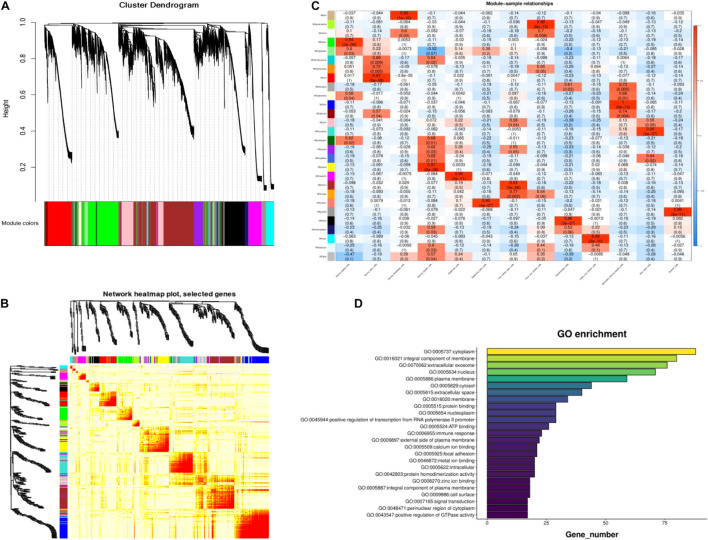
Module identification of cashmere goat skin. **(A)** Cluster dendrogram of all screened genes. **(B)** TOM of all screened genes. **(C)** Heat map of the correlation between modules and cashmere skin cells. Each specific color represents the specific gene module. **(D)** GO analysis of the core genes in blue module.

To further identify the genes related to cashmere growth, we conducted Weighted gene correlation network analysis (WGCNA) on cashmere goat skin. WGCNA, a relatively new statistical analysis, is usually used to construct networks based on gene correlations and recognize intramodular hub genes (Tahmasebi et al., 2019). The average expression profile of each gene in each type of cell was analyzed to screen genes for WGCNA. The cluster dendrogram consists of 31 coexpression modules, including tan, green, yellow, white, green, and other colors (Figure 3A). We also found that, compared with others, the blue module had a strong coexpression relationship by building a topological overlap matrix (TOM) diagram (Figure 3B). The darker the color, the higher the topological overlap.

As shown in Figure 3C, all genes in the 13 type cells were selected to produce the module trait relationship. Interestingly, before, there was almost no similarity between each module, suggesting that most modules had a certain promoting effect on each cluster. The significant enrichment module in SDPCs is blue, in DPCs is green, and in stromal cells is gray60, and the number of genes per module was 2,629, 1,839, and 256, respectively. The blue module of the SDPC cluster related to cashmere growth had the deepest color, suggesting that genes in this module might have a positive effect on cashmere fineness. We identified hub genes *COL1A1, COL1A2*, *CCL26*, *CCDC80*, *HOXA13*, *CXCL8*, *KRT24*, and *BMP3* as having significant connectivity, which may be associated with cashmere development.

Enrichment analysis was used to explore the biological functions of genes in these modules to further determine the relationship between these genes and cashmere growth. The GO analysis results of the core genes in the blue module are displayed in Figure 3D. It was found that these genes are suggestive of cytoplasm, an integral component of the membrane.

**FIGURE 4:**
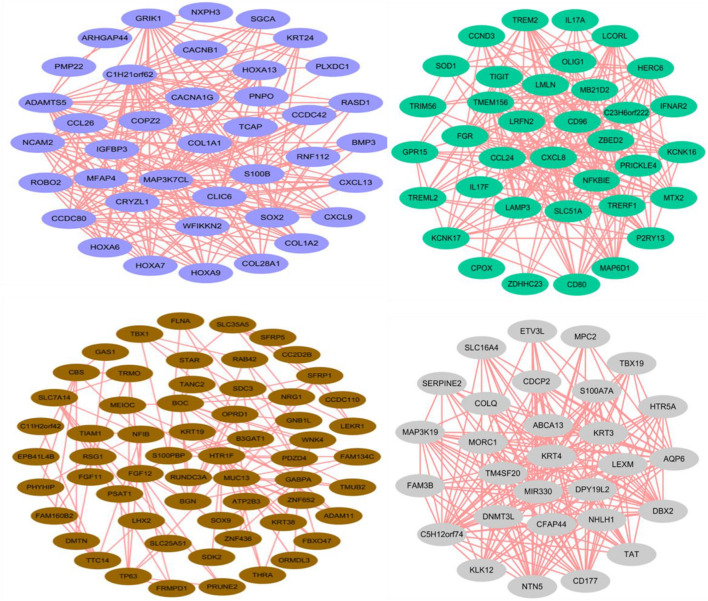
Coexpression networks of core genes in blue, brown, green, and gray60 modules. Each color represents a module.

Through the correlation analysis between modules and samples, we selected four modules with the highest correlation to construct the network diagram. The detailed network information of the core genes for four selected modules, including blue, brown, green, and gray60 modules, is shown in Figure 4. In the gene networks, some hub genes interacted with many other genes, which indicate that they are more likely to be necessary. *COL1A1*, *CCDC80*, and *HOXA13* are associated with many genes in the blue module. *CXCL8*, *NFKBIE*, and *CCL24* are active in the green module. *KRT3*, *KRT4*, and *KLK12* are positively linked to other genes in the gray60 module. *KRT19*, *KRT38*, *NFIB*, and *LHX2* are positive genes in the brown module. *COL1A1*, with a significant connectivity in SDPCs, may serve as a target for cashmere formation.

### Identification and Analysis of Dermal Papilla Cells and Secondary Hair Follicle Dermal Papilla Cells in Cashmere Goat Skin

**FIGURE 5:**
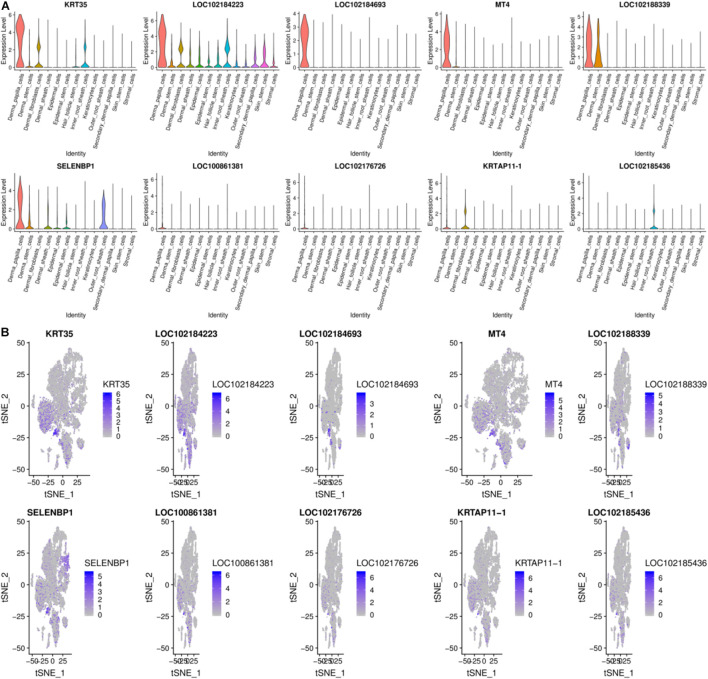
Gene expression profiling of DPCs. **(A)** Violin plots for expression of top 10 genes. **(B)** Relative expression levels of top 10 genes on the t-SNE plots.

Previous work shows that DPCs can induce hair follicle regeneration, determine the size of hair follicles to a certain extent, and play an essential role in the hair follicle cycle change process (Ma et al., 2019). Moreover, DPCs are important signaling components that guide the proliferation, upward migration, and differentiation of HF stem cell progenitor cells to form other types of HF cells (Dai et al., 2021). DPCs and SDPCs have different effects on the growth of hair: DPCs control the growth of wool, and SDPCs decide the cashmere (Shen, 2017). DPCs subtly grasp the continuous growth of mammalian hair, including cashmere. There are 100 high-expression genes in DPCs, and the top10 genes from 13 cell clusters are exhibited in Figure 5. *LOC102184693*, *KRT35*, *KRTAP11-1*, *KRTAP3-1*, and *MT4* almost only expressed in DPCs. In hair follicle and goat skin, *KRT35* and *KRTAP11-1* frequently appear. Moreover, we detected two crucial activators *HOXC8* and *RSPO1* that were enriched in DPCs as reported (Ma et al., 2019). In addition, the family of RPS and RPL are significantly expressed in DPCs, such as *RPS8*, *RPS2*, *RPL0*, and *RPL32*. *RPS12*, as the key effector of cell competition caused by other RP gene mutations, has the highest expression in DPCs. Among them, *PTGER4* and *ESR1* are more abundant in DPCs consistent with previous studies (Movérare et al., 2002; Torii et al., 2002). To better understand the functioning approaches of DEGs in DPCs, GO, and KEGG enrichment analysis were performed. Finally, 452 pathways were enriched, such as keratin filament and hair follicle morphogenesis. By KEGG analysis, some pathways related to cashmere growth and development were enriched, including TGF-beta signaling pathway, alcoholism, PPAR, and Notch signaling pathway.

**FIGURE 6:**
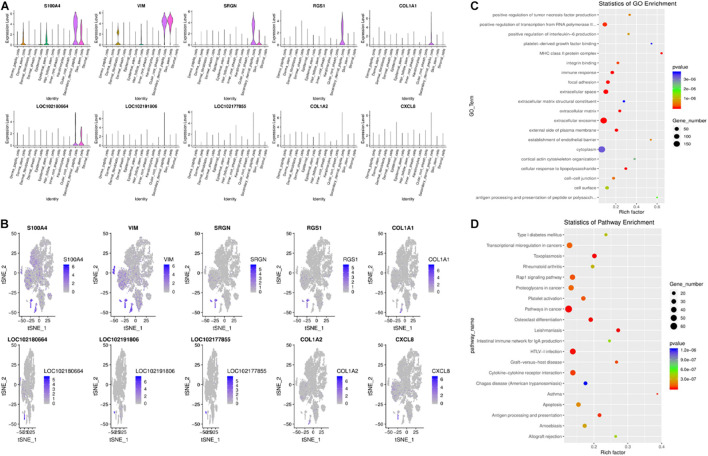
Gene expression profiling of SDPCs. **(A)** Violin plots for expression of top 10 genes. **(B)** Relative expression levels of top 10 genes on the t-SNE plots. **(C)** Top 20 significantly enriched GO terms. **(D)** Top 20 KEGG pathways.

Recently, research in this field has indicated that hair type can be determined by SDPCs. In SDPCs, the top10 up-expression genes are exhibited in Figures 6A,B. Of note, *COL1A1*, *COL1A2*, *COL3A1*, *S100A4*, *VIM*, *CRABP2*, *CCDC80*, *ACTG2*, *CCL26*, and *CXCL12* are solely abundant in SDPCs. *COL1A2*, *COL3A1*, and *CCDC80* are reported to be differentially expressed in different cashmere fibers (Zheng et al., 2019). Furthermore, the expression of *S100A4* was the highest in SDPCs, which may involve Yangtze River Delta white goat hair growth (Li et al., 2013). Also, *NFKBIA* is highly enriched in SDPCs, which may play an important role in cashmere growth (Jin et al., 2018; Zheng et al., 2019). Similarly, *CRABP1*, a constant marker of DPCs, has a high expression in SDPCs and expresses throughout all stages of hair cycling (Driskell et al., 2012). To further excavate the critical genes regulating cashmere fineness, enrichment was analyzed in SDPCs. A total of 848 GO terms were significantly enriched; as expected, hair follicle morphogenesis, hair follicle development, and intermediate filament cytoskeleton were significantly enriched GO terms. The top 20 differentially expressed GO terms are shown in Figure 6C. The top 20 pathways by KEGG analysis were significantly enriched, such as PI3K-Akt, TGF beta, and WNT signaling pathways (Figure 6D). *BMP3*, *BMP4*, *BMP7*, *FGF7*, *FGF2*, *COL1A1*, *IGF1*, and *WNT5A* enriched these pathways.

**FIGURE 7:**
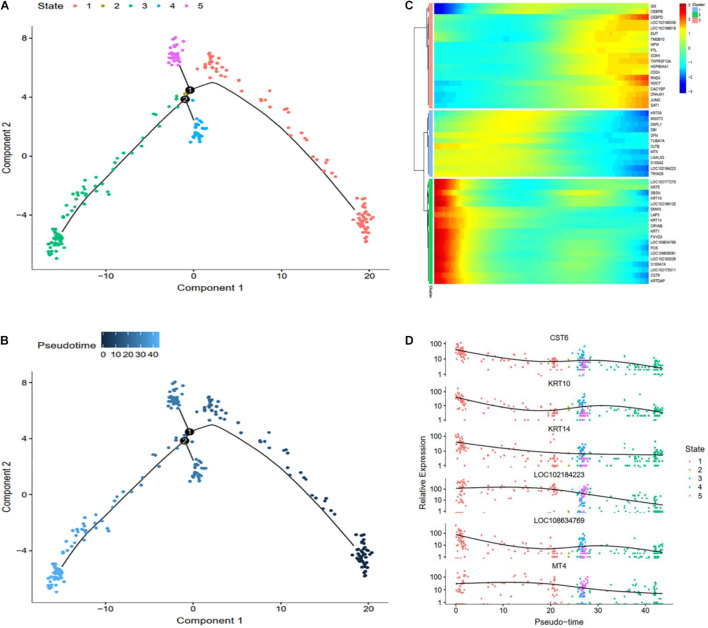
Cell trajectory analysis in DPCs of goat skin. **(A)** Monocle 2 cell trajectory of goat DPCs. **(B)** Pseudo-time is displayed in a dark to light blue gradient and indicates the start of pseudo-time. **(C)** Clustering of top 50 genes exhibiting in DPCs. **(D)** Expression levels of six responsive genes in DPCs. *X*-axis represents pseudo-time, *Y*-axis represents gene expression levels.

**FIGURE 8:**
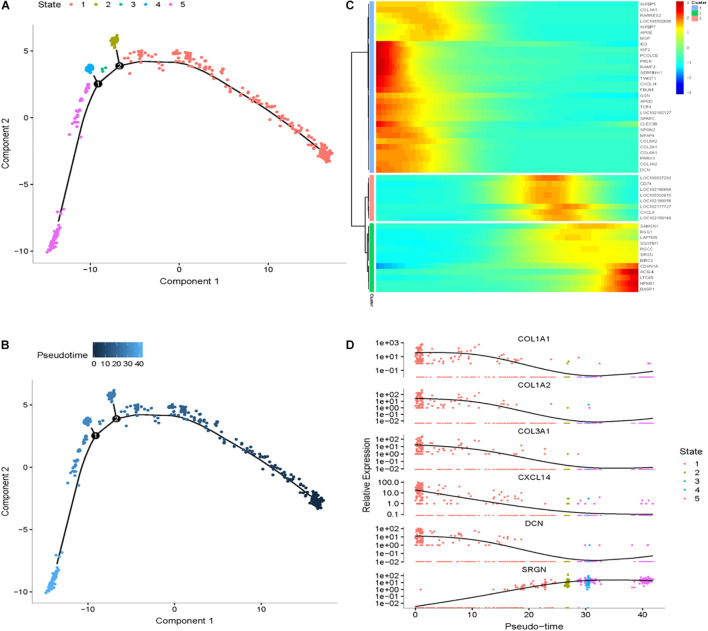
Cell trajectory analysis in SDPCs of goat skin. **(A)** Monocle 2 cell trajectory of goat DPCs. **(B)** Pseudo-time is displayed in a dark to light blue gradient and indicates the start of pseudo-time. **(C)** Clustering of top 50 genes exhibiting in SDPCs. **(D)** Expression levels of six responsive genes in DPCs. *X*-axis represents pseudo-time, *Y*-axis represents gene expression levels.

To explore differential genes in the development period, we took a closer look at cell trajectory analysis of DPCs and SDPCs, respectively. Trajectory analysis provided the development process and five differentiation states in DPCs (Figures 7A,B). *CST6*, *KRT10*, *KRTDAP*, and *DMKN* decreased first, then rose and fell at DPCs (Figures 7C,D), and yet *KRT35* and *MT4* were momentarily up-regulated and then down-regulated. *SOX4* and *CEBPB* were continually up-regulated, and *KRT14* and *KRT1* were down-regulated. Cell trajectory analysis demonstrated a total of five states in SDPC development stages (Figures 8A,B). *CXCL8* and *CD34*, after a transient period of stability, began to down-regulate (Figure 8C). Notably, *CXCL4*, *COL1A1*, *COL1A2*, and *COL3A1* were continually downregulating, and *SRGN*, *NFKB1*, and *RGCC* showed late specific expression (Figure 8D). As expected, GO analysis showed that DPCs and SDPCs both significantly enriched *KRT14, CXCL4*, and *COL1A1* in the development process, and with the development of a pseudo-timeline on the axis, these genes involved in the development were down-regulated. *KRT35*, *SRGN*, and *NFKB1* showed specific expressions in development later. *CXCL4*, *COL1A1*, *KRT14*, and *KRT35* may be the key candidate genes for regulation DPCs and SDPCs.

### Analysis and Verification of Genes Difference in Secondary Hair Follicle Dermal Papilla Cells of CT-LCG and FT-LCG

**FIGURE 9:**
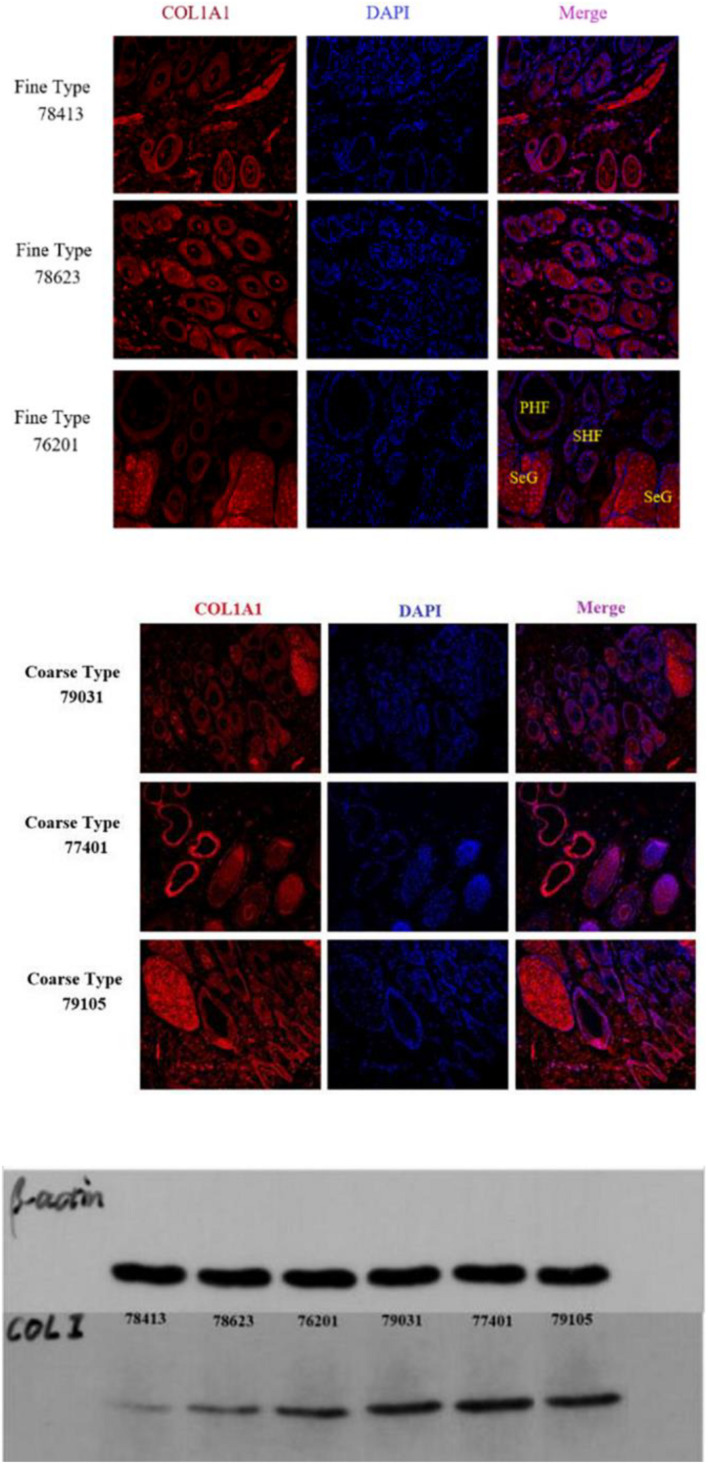
IF results of *COL1A1* in FT-LCG and CT-LCG skin tissues (200x).

**FIGURE 10:**
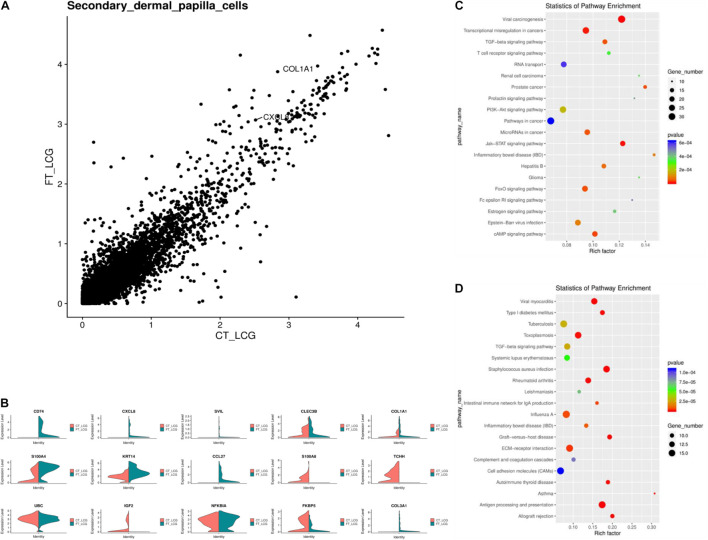
Analysis of DEGs between CT-LCG and FT-LCG SDPCs. **(A)** Scatterplot of DEGs in SDPCs. **(B)** Top 20 up-expressed genes in SDPCs. Red: CT-LCG; Green: FT-LCG. **(C)** KEGG analysis in CT-LCG. **(D)** KEGG analysis in FT-LCG.

IF results of COL1A1 in FT-LCG and CT-LCG skin tissues (200x) (Figure 9). A total of 16,601 genes were found in SDPCs of two samples (Figure 10A), and there are 15 representative DEGs between CT-LCG and FT-LCG exhibited in Figure 10B. *TCHH*, *S100A8*, and *IGF2* were highly expressed in CT-LCG, and CD74, CCL27, *COL1A1*, and *CXCL8* were highly expressed in FT-LCG. To further excavate the critical genes regulating cashmere fineness, enrichment was analyzed in SDPCs. There are 48 and 45 significant pathways in CT-LCG and FT-LCG. CT-LCG are mainly enriched to the Jak-STAT, TGF-beta, and PI3K-Akt signaling pathways (Figure 10C), and FT-LCG is also enriched in the TGF-beta signaling pathway (Figure 10D). *COL1A1* is enriched in the PI3K-Akt signaling pathway, and *CXCL8* enriched to the NF-kappa B signaling pathway, which is reported to may be related to cashmere. We found that there were significant differences in the highly expressed genes and enrichment pathways in CT-LCG and FT-LCG.

**FIGURE 11:**
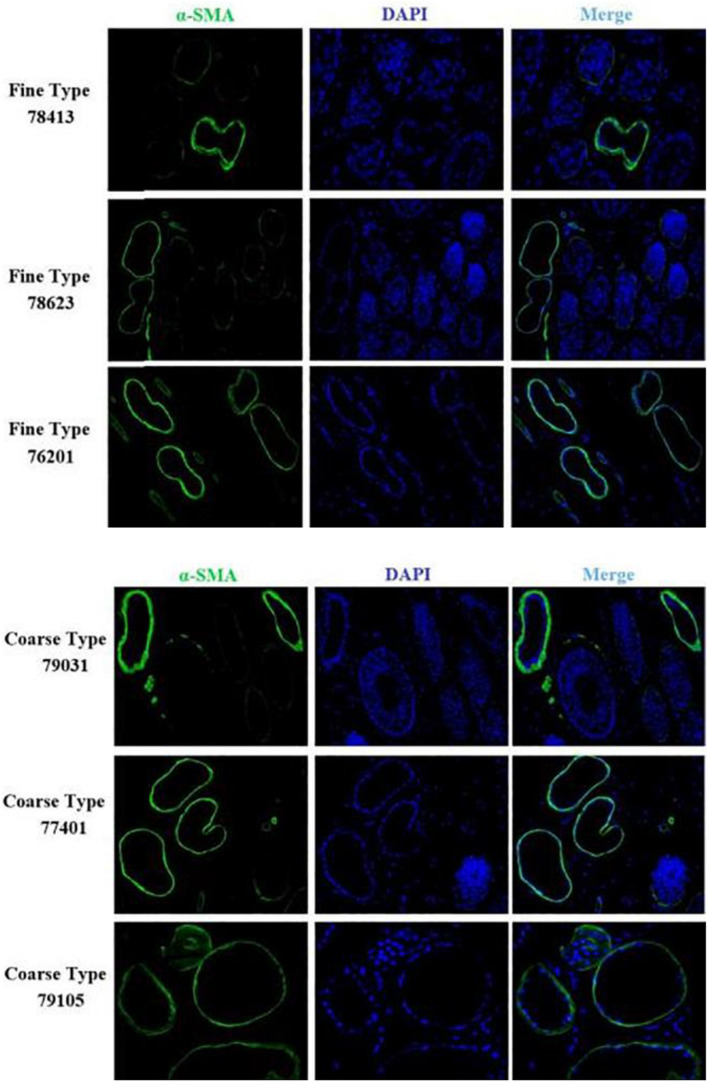
IF results of *ACTA2* (*α-SMA*) in FT-LCG and CT-LCG skin tissues (200x). *ACTA2* is basically expressed in SHFs.

**FIGURE 12:**
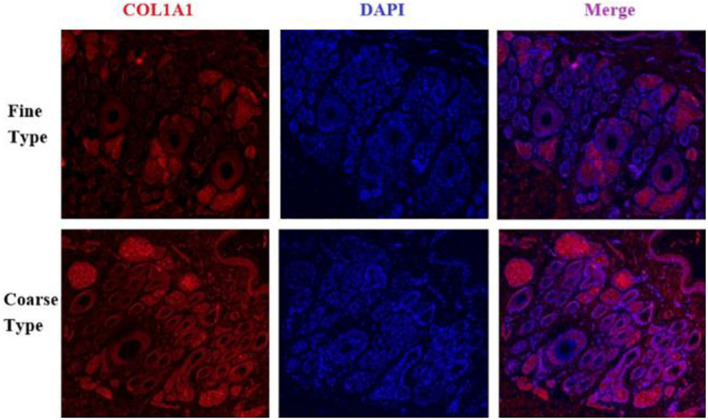
IF results of *COL1A1* in FT-LCG and CT-LCG skin tissues (40x).

**FIGURE 13:**
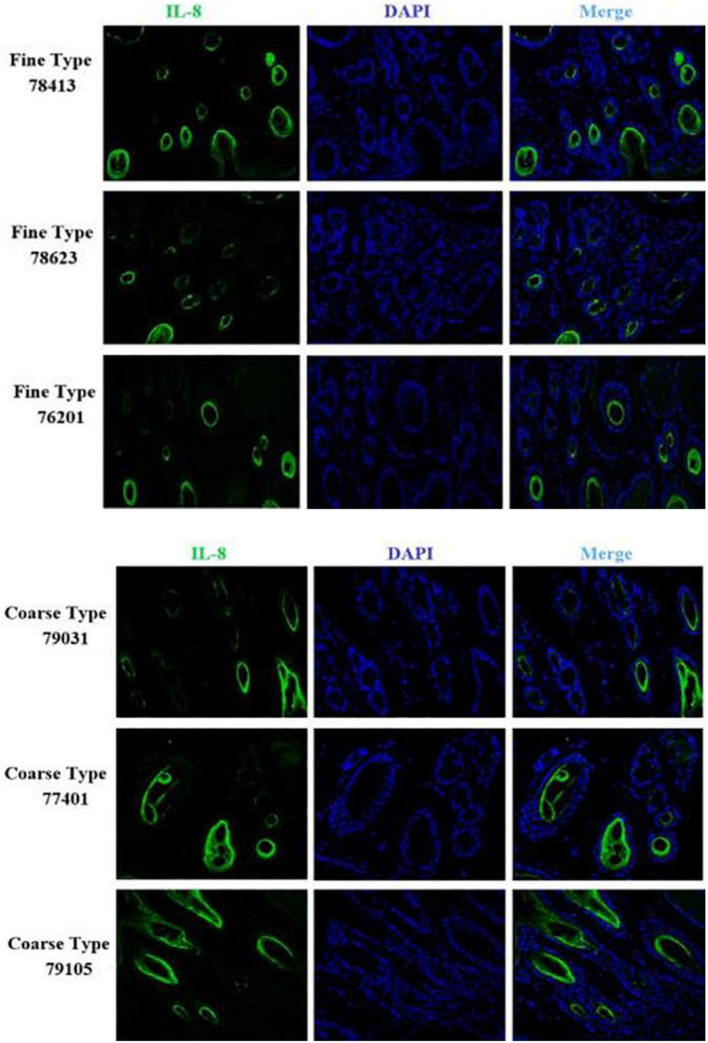
IF results of *CXCL8* in FT-LCG and CT-LCG skin tissues (200x).

We identified ACTA2 (α-SMA) as a marker gene of SDP cells by IF, and α-SMA protein was almost exclusively expressed on secondary hair follicles as shown in Figure 11. Meanwhile, we also chose COL1A1 and CXCL8 as marker genes of SDP cells. To further determine the relationship between these genes and cashmere fineness, we validated COL1A1 and CXCL8 in SDPCs by IF assay. We conducted IF tests on the skin of three kinds of coarse hairs and three kinds of fine hairs of Liaoning cashmere goats. The differential expressions of COL1A1 in coarse- and fine-type skin indicate that the number of SHFs in fine skin is more distributed, arranged tightly, and with smaller cashmere diameter, and the number of SHFs in coarse skin is less distributed, arranged sparsely, and with larger cashmere diameter (Figure 12). Wang shows that COL1A1 has differences in CT-LCG and FT-LCG (Jin et al., 2018). In the results of IF, the nucleus is blue, and the target protein is green. PHF is the primary hair follicle, and SHF is the secondary hair follicle, which is the place where cashmere grows. SeG is sebaceous glands. They form the hair follicle cluster. The SDPCs existed in the SHFs. CXCL8 is the DEG of CT-LCG and FT-LCG screened by us. The results show that the expression level of CXCL8 (IL-8) protein in CT-LCG was lower than that in FT-LCG (Figure 13). The results of fine and coarse fluorescent immunoassays are presented below. From the results, we can see that the results of IF are consistent with the results of single-cell sequencing.

**FIGURE 14:**
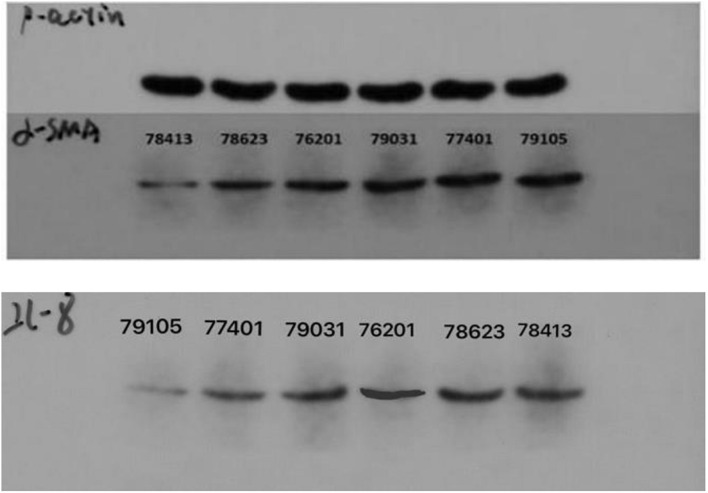
The electrophoretogram of *ACTA2 (α-SMA)* and *CXCL8 (IL-8)* at CT-LCG and FT-LCG.

The optical density (OD) value of protein standard was measured at the wavelength of 568 nm, and the standard curve was established. The regression equation and variance coefficient were *y* = 0.6965*x* + 0.0141 and *R*^2^ = 0.997. Meanwhile, the results of electrophoresis show that the expression level of ACTA2 (α-SMA) and CXCL8 in CT-LCG was lower than that in FT-LCG as shown in Figure 14.

## Discussion

Cashmere goats mainly produce cashmere fiber, which plays an indispensable role in the textile industry. Cashmere fineness, the characteristic of natural quality, is a very important evaluation index in cashmere production. The study on cashmere diameter is the core issue of cashmere industry development. However, research on the fineness of cashmere is limited, and there is almost no research on the cellular level. The development of sscRNA-seq not only enriches the research methods of identifying cell types in cashmere goat skin, but also provides more candidate genes for hair formation regulation. Previous studies use single cell sequencing technology to reveal the molecular signatures of human, mouse, and other animals (Guo et al., 2018; Tepe et al., 2018; Fan et al., 2019; Zhang et al., 2020).

Here, we use single-cell sequencing to recognize 13 clusters of cells in Liaoning cashmere goat skin, including DPCs, SDPCs and stromal cells. We detected highly expressed genes in every cell type, and the development process of goat skin was studied by cell trajectory analysis. Differential gene expression profiles of 13 clusters provide a unique opportunity to identify positive markers to study the function of rare and insufficient cashmere goat skin. Then, we identified 31 modules in 13 types of cells through WGCNA, and the blue module was the most significant one in the SDP related to cashmere growth. Finally, we identified 16,601 genes, and 15 typical DEGs were screened in SDPCs between CT-LCG and FT-LCG, and *COL1A1* and *CXCL8* were verified. Our current study provides the first step to fill in the knowledge gaps about the characteristics of skin cell types in cashmere goats.

The cell trajectory analysis of cashmere goat skin is divided into 15 stages and seven nodes. With the development of the time axis, *KRTAP11-1*, *KRTDAP*, and *TCHH* play a role in early development, and *KRT5*, *PPS7*, and *RPL5* play a role in development later. The results show that most of these genes were active in the later stage of skin development. GO analysis enriched to intermediate filament and keratinization, and KEGG pathway analysis mainly enriched to WNT, TGF beta, and PI3K-Akt signaling pathways, indicating that these genes may relate to cashmere formation and development. The WGCNA results have enriched 31 modules in total, and we have carried out network analysis on four modules. *COL1A1*, *CCDC80*, *HOXA13*, *NFKBIE*, *CCL24*, *KRT3*, *KRT4*, *KRT19*, *KRT38*, *NFIB*, and *LHX2* are the key genes in the co-expression analysis module. *COL1A1* is an important gene of the result of pseudo-time analysis and WGCNA in SDPCs of cashmere goat, which may be related to cashmere formation. In the clustering analysis and cell trajectory analysis, we removed batch effects by harmony.

Dermal papilla cells act as a relay station to transmit the effects of hormones and other molecules produced locally or systematically on hair growth (Paus et al., 2014), which also can instruct the activity of follicular keratinocytes to reshape hair follicles and produce a new hair shaft. Also, previous studies demonstrate that the induction characteristics of DPCs play a key role in morphogenesis, development, and hair formation of hair follicles and regulates the hair growth cycle after birth (Yang and Cotsarelis, 2010; Driskell et al., 2012). It is well known that some characteristic molecules are considered as classic signs to evaluate the induction characteristics of DPCs, such as *HSPC016*, *Wnt10b*, *BMP6*, and *Wnt5a* (Hu et al., 2010; Yang and Cotsarelis, 2010; Ouji et al., 2012; Song et al., 2012). However, their roles in the regulation of DPC secretion and their molecular mechanisms are still unclear. In our study, we observed that *KRT35*, *KRTAP11-1*, and *KRTAP3-1* have unique expressions in DPCs. *SOX2*, *WNT10b*, *HOXC8*, and *RSPO1* also have high expressions in DPCs. *SOX2* is a specific marker gene in DPCs and its restricted expression in guard (Driskell et al., 2012). *Wnt10b* can accelerate the proliferation of DPCs and also has a potent ability to maintain *VERSICAN* expression in DPCs and sustained hair follicle (Ouji et al., 2012). Additionally, Ma defined 25 core signatures of cashmere goat DPCs, such as *HOXC8* and *RSPO1* (Ma et al., 2019).

Recently, a considerable number of genes have been identified that might be involved in reconstruction and development of cashmere goat SHFs. Wang reports some genes, such as *HOXC13*, *SOX9*, *JUNB*, *LHX2*, and *GATA3*, involved in hair follicle differentiation through selective expression in embryonic day 120 (Wang et al., 2020). In recent years, studies about cashmere goat SDPCs have increased (Geng et al., 2013; Mitoma et al., 2014; Liu et al., 2016; Yang et al., 2017); however, little information was available associated with the development and cashmere fineness of Liaoning cashmere goat SDPCs. In our investigations, we demonstrate that some genes exhibited significantly different expressions in SDPCs, such as *COL1A1*, *COL3A1*, and *CCDC80*. *S100A4* had the highest enrichment in SDPCs, which were discovered in the human hair follicle DPCs (Mitoma et al., 2014). In addition, we identified 15 DEGs in two kinds of fiber fineness Liaoning cashmere goat skin, and *CXCL8* had a significant difference in SDPCs of CT-LCG and FT-LCG. SDPCs play an important role in the growth and density of cashmere.

In conclusion, we identified 13 kinds of cells and DEGs in the skin of cashmere goats. The skin development of cashmere goats can be divided into 15 stages through the analysis of the cell trajectory. According to the network constructed by WGCNA, 31 modules were determined, and four modules were selected for detailed analysis, among which the genes in the blue module were the most significant in SDPCs. The results of scRNA-seq and IF demonstrate that *COL1A1* may act in the regulation skin development, and *CXCL8* may regulate cashmere fineness in Liaoning cashmere goats. Furthermore, our study provides valuable resources for the identification of a cell map of formerly unknown cashmere goats, which, in turn, will help to study the special contribution of cells to cashmere fiber.

### Potential Implications

Cashmere fineness is one of the important factors determining cashmere quality; however, our understanding about the cells that make up the cashmere fineness is limited. We identified the key genes regulating cashmere formation in cashmere goat skin by 10 × Genomics single-cell sequencing. These genes may be involved in regulating the fineness of cashmere in goat SDPCs, and our research will provide new insights into the mechanism of cashmere growth and cashmere fineness regulation by cells.

## Materials and Methods

### Tissue Dissociation and Preparation of Single-Cell Suspensions

The skin tissue of cashmere is taken from the scapularis of two healthy Liaoning cashmere goats from the Liaoning Animal Husbandry Research Institute. The cashmere fineness of the two goats is 17.19 and 14.3 μm. Place a sterile RNase-free culture dish containing an appropriate amount of calcium-free and magnesium-free 1 × PBS on ice, the tissue was transferred into the culture dish and cut into 0.5-mm^2^ pieces, the tissues were washed with 1 × PBS to remove as many nonpurpose tissues as possible, such as blood stains and fatty layers. Tissues were dissociated into single cells in dissociation solution (0.35% collagenase IV5, 2 mg/ml papain, and 120 units/ml DNase I) in a 37°C water bath with shaking for 20 min at 100 rpm. Digestion was terminated with 1 × PBS containing 10% fetal bovine serum (FBS, V/V), then pipetting 5–10 times with a Pasteur pipette. The resulting cell suspension was filtered by passing through 70–30 μm stacked cell strainer and centrifuged at 300 g for 5 min at 4°C. The cell pellet was resuspended in 100 μ l 1 × PBS (0.04% BSA) and added to 1 ml 1× red blood cell lysis buffer (MACS 130-094-183, 10×) and incubated at room temperature or on ice for 2–10 min to lyse the remaining red blood cells. After incubation, the suspension was centrifuged at 300 g for 5 min at room temperature. The suspension was resuspended in 100 μl Dead Cell Removal MicroBeads (MACS 130-090-101) to remove dead cells using Miltenyi^®^ Dead Cell Removal Kit (MACS 130-090-101). Then the suspension was resuspended in 1× PBS (0.04% BSA) and centrifuged at 300 g for 3 min at 4°C (repeat twice). The cell pellet was resuspended in 50 μl of 1× PBS (0.04% BSA). The overall cell viability was confirmed by trypan blue exclusion, which needed to be above 85%. Single cell suspensions were counted using a Countess II Automated Cell Counter and concentration adjusted to 700–1,200 cells/μl. All experimental procedures used in this study were approved and conducted according to the guidelines by the Laboratory Animal Management Committee of Shenyang Agricultural University; animal welfare number is 201806019.

### Chromium 10 × Genomics Library and Sequencing

Single-cell suspensions were loaded on to 10x chromium to capture single cells according to the manufacturer’s instructions of the 10X Genomics Chromium Single-Cell 3’ kit (V3). The following cDNA amplification and library construction steps were performed according to the standard protocol. Libraries were sequenced on an Illumina NovaSeq 6000 sequencing system (paired-end multiplexing run, 150 bp) by LC-Bio Technology Co. Ltd. (Hangzhou, China) at a minimum depth of 20,000 reads per cell.

### Bioinformatics Analysis

We used 10x Genomics official analysis software Cell Ranger^1^ to filter, compare, quantify, and identify the recovered cells, and finally get the gene expression matrix of each cell. Seurat^2^ was used for further cell filtration, standardization, and classification of cell subsets; DEG analysis of each subgroup; and marker gene screening. We performed cell filtration based on the number of genes expressed by cells (using the gene expression number 500 as the threshold). The filtering of low-quality cells uses the Seurat data analysis R package. Its functions include quality control, filtering, data standardization, principal component analysis (PCA), t-SNE differential gene analysis, etc. At the same time, it has the function of visualizing the analysis results (Butler et al., 2018). Seurat analyzed the DEGs in different cell populations by the bimod (McDavid et al., 2013) likelihood ratio statistical test to screen up-regulated genes in different cell populations.

To visualize the data, we further reduced the dimensionality of all 13,051 cells using Seurat and used t-SNE to project the cells into 2-D space. The steps include (1) using the LogNormalize method of the “Normalization” function of the Seurat software to calculated the expression value of genes; (2) PCA was performed using the normalized expression value. Within all the PCs, the top 10 were used to do clustering and t-SNE analysis. (3) To find clusters, select the weighted shared nearest neighbor graph-based clustering method. Marker genes for each cluster were identified with bimod with default parameters via the find all markers function in Seurat (version 3.1.1). This selects marker genes that are expressed in more than 10% of the cells in a cluster and average log (fold change) of greater than 0.26. For cell trajectory analysis, we used the Monocle analysis toolkit (Trapnell et al., 2014), and Monocle 2 analysis was used to order cells in pseudo-time. We use the default function to estimate the size factor and dispersion. We applied DDRTree for dimension reduction. To visualize all pseudo chronos genes that may regulate cashmere development, we cluster the regulation of genes according to time changes. GO^3^ and KEGG are the functions of Kyoto Gene and Genome Encyclopedia^4^ to analyze the function of differential genes. The results are displayed using a ggpl set analysis scatterplot. GO/KEGG Bioinformatic analysis was performed using the OmicStudio tools at https://www.omicstudio.cn/tool.

### Weighted Gene Correlation Network Analysis

The co-expression network was performed using the “WGCNA” R package for the filtered genes (Langfelder and Horvath, 2008). We use the pairwise Pearson coefficient to find the co-expression adjacency matrix of all genes in the weighting. The TOM matrix is used to estimate its connectivity in the network (Langfelder and Horvath, 2012). The co-expression network analysis of hub gene in modules was established by Cytoscape 3.5.1 software. Each node represents a gene associated with a different number of genes. The partition module uses the BlockWiseModules function in the WGCNA package. The main parameters are power = 9; merge cut height = 0.25; everything else is default; the network graph visualization uses the Export Network to Cytoscape function in the WGCNA package. The main parameter: threshold = 0.1, and the other parameters are the default parameters.

### Immunofluorescence

Skin tissue used for this test was derived from six Liaoning cashmere goats, including three fine-type goats and three coarse-type goats. The goat number of the three fine goats were 78,413, 78,623, and 76,201, and their cashmere fineness is 14.32, 14.69, and 14.77 μm, respectively. The goat number of the three coarse goats is 79,031, 77,401, and 79,105, and their cashmere fineness is 17.23, 17.63, and 17.91 μm, respectively. The skin tissues were fixed with 4% paraformaldehyde for 24 h, dehydrated with gradient alcohol, treated with xylene transparently, and embedded in paraffin. Slides were sliced by a Leica pathological slicer (Leica RM 2016 rotary slicer, Germany). The slides were put into a 40°C water bath pot for spreading. The anti-stripping glass piece is inserted into the water surface obliquely to remove the slice so that the slice is attached to the appropriate position of the slides. The slice was baked in a 60°C oven (Wuhan Junjie jk-6 biological tissue spreading and baking machine) for 3 h. The paraffin sections were successively put into xylene I (20 min) – xylene II (20 min) – xylene III (20 min) – anhydrous ethanol I (5 min) – anhydrous ethanol II (5 min) – 95% alcohol (5 min) – 90% alcohol (5 min) – 80% alcohol (5 min) – 70% alcohol (5 min), and then soaked in distilled water for 5 min. Microwave was used for antigen repair. The dewaxed and hydrated tissue sections are placed on the high temperature–resistant plastic section frame in the beaker (or repair box), and a proper amount of repair solution (0.01 M citric acid buffer, pH 6.0) is added in the beaker. The liquid surface should be soaked in the slides to a certain height. The microwave oven can first use high-grade heating to make the liquid boil. When heating to boiling, adjust it to the middle level and start timing; the repair time is 15 min. Take out the beaker from the microwave oven, put it into cold water to cool down, take out the glass slide when the repair solution drops to room temperature, wash it with PBS (pH 7.4) three times, each time for 3 min. Dry the slides with absorbent paper, draw a circle around the tissues with the brush of the immune group, drip diluted normal goat serum, and seal it at room temperature for 30 min to reduce nonspecific staining. Shake off the excess liquid, do not wash it, then drop the diluted first antibody, after adding the first antibody, incubate it in a 4°C wet box overnight (15 h). The sections were washed by BST three times for 3 min each time. After the sections were dried by absorbent paper, the diluted fluorescent secondary antibody was added. The slides were incubated in a wet box at 37°C for 1 h. The slides were washed by PBST four times, 3 min each time. The sections were washed by BST three times, 3 min each time. After the sections were dried by absorbent paper, the diluted fluorescent secondary antibody was added. The sections were incubated in a wet box at 37°C for 1 h. The sections were washed by PBST four times, 3 min each time. Primary antibody: collagen I (Wuhan doctoral Bioengineering Co., Ltd.), ACTA2 (Abcam), IL-8 (Abcam), dilution ratio is 1:100. Secondary antibody: fluorescence (Cy3) labeled Sheep anti rabbit IgG (Abcam), fluorescence (Cy3) labeling of Goat anti mouse IgG, dilution ratio is 1:100.

### Western Blot

The proteins in each group were extracted using the radio immunoprecipitation assay lysis buffer added with protease inhibitors. Then, quantification of protein was performed with a bicinchoninic acid Protein Assay Kit. The extracted protein supernatant was mixed with 5× sodium dodecyl sulfate (SDS)-polyacrylamide gel electrophoresis (PAGE) loading buffer (the volume ratio was 4:1) and put into boiling water for 10 min. Prepare electrophoresis gel, which consists of 6 ml 5% concentrated gum and 20 ml 12% separating gum, the former consists of 4.1 ml of H_2_O, 1.0 ml 30% Acrylamide, 0.75 ml 1.0 Mtris–HCl (pH6.8), 0.06 ml 10% SDS and 0.06 ml APS; the latter consists of 6.6 ml of H_2_O, 8.0 ml 30% Acrylamide, 5.0 ml 1.5 Mtris–HCl (pH8.8), 0.2 ml 10% SDS, 0.2 ml APS, and 0.008 ml TEMED. Fix the prepared glue on the electrophoresis tank and pour the electrophoresis solution into the storage tank. The prepared protein sample and marker were added into the sample hole with a micro sampler, and the total protein content of each sample was 40 μg. After adding the sample, first constant pressure 80 V electrophoresis was applied to the Bromphenol blue indicator at the junction between the concentrated and separation glue and changed to constant pressure 120 V to Bromphenol blue to the bottom of gel; this process is about 1.5 h. Take out the gel, cut the target strip according to marker, rinse with distilled water, cut the same size polyvinylidene fluoride (PVDF) film and filter paper with the PAGE gel, immerse the PVDF membrane with methanol for a few seconds, and immerse it in the electromigration buffer with the filter paper. According to the black plate – fiber mat – filter paper – gel – PVDF membrane – filter paper – fiber mat – white plate in good order, after clamping the plate into the rotating film instrument, black plate side contrast black negative pole. Fill the membrane transfer tank with electric fluid to start membrane transfer. The PVDF membrane was soaked in tris buffered saline tween (TBST; blocking solution) containing 5% skimmed milk powder and sealed in a shaker at room temperature for 2 h. The PVDF membrane was immersed in the primary antibody incubation solution and incubated overnight at 4°C. The antibodies are β-actin, Collagen I, ACTA2, and CXCL8. Their dilution is 1:1000. The PVDF membrane was fully washed by TBST five times, 5 min/time. Put up to three films in a dish, and pay attention to whether the film is attached to the dish wall or whether the films overlap during membrane washing. TBST was used to dilute the corresponding HRP labeled second antibody—1:50,000, and the PVDF membrane was immersed in the second antibody incubation solution and incubated in a shaker at room temperature for 2 h. The PVDF membrane was fully washed by TBST five times, 5 min/time. Mix the reinforcement solution and stable peroxidase solution of electrochemiluminescence reagent in the proportion of 1:1, drop the working solution onto PVDF membrane, react for several minutes, after the fluorescence band is obvious, use filter paper to absorb the excess substrate solution, cover with fresh-keeping film, press the X-ray film, then successively add the developing solution, fixing solution, and develop the film. Dry the film, scan the film, and analyze the gray value of the film with IPP. β-actin was used as a loading control.

## Data Availability Statement

The datasets presented in this study can be found in online repositories. The names of the repository/repositories and accession number(s) can be found here: GEO GSE182474.

## Ethics Statement

The animal study was reviewed and approved by the Laboratory Animal Management Committee of Shenyang Agricultural University.

## Author Contributions

ZW and RW design the project. YYZ, YRW, TH, and RC carry out the experiment. YXZ, XD, XHZ, and DG provide resources. JS, WC, XJZ, MG, and YQ perform and isolate sample collection. YYZ, YRW, TH, YX, YZ, SG, CY, and ZB perform the bioinformatic analysis. HT, WW, YC, MB, and YF perform the soft. All authors reviewed the manuscript.

## Conflict of Interest

The authors declare that the research was conducted in the absence of any commercial or financial relationships that could be construed as a potential conflict of interest.

## Publisher’s Note

All claims expressed in this article are solely those of the authors and do not necessarily represent those of their affiliated organizations, or those of the publisher, the editors and the reviewers. Any product that may be evaluated in this article, or claim that may be made by its manufacturer, is not guaranteed or endorsed by the publisher.

## Footnotes

1
https://support.10xgenomics.com/single-cell-geneexpression/software/overview/welcome

2
https://satijalab.org/seurat/

3
http://geneontology.org/

4
www.genome.jp/kegg
